# Supplementary figures and images for: Cancer stage at presentation for incarcerated patients at a single urban tertiary care center
Source: PLoS One. 2020 Sep 15;15(9):e0237439. doi: 10.1371/journal.pone.0237439 (PMC7491712; doi:10.1371/journal.pone.0237439)

**S1 Fig.** Construction of the Study Sample


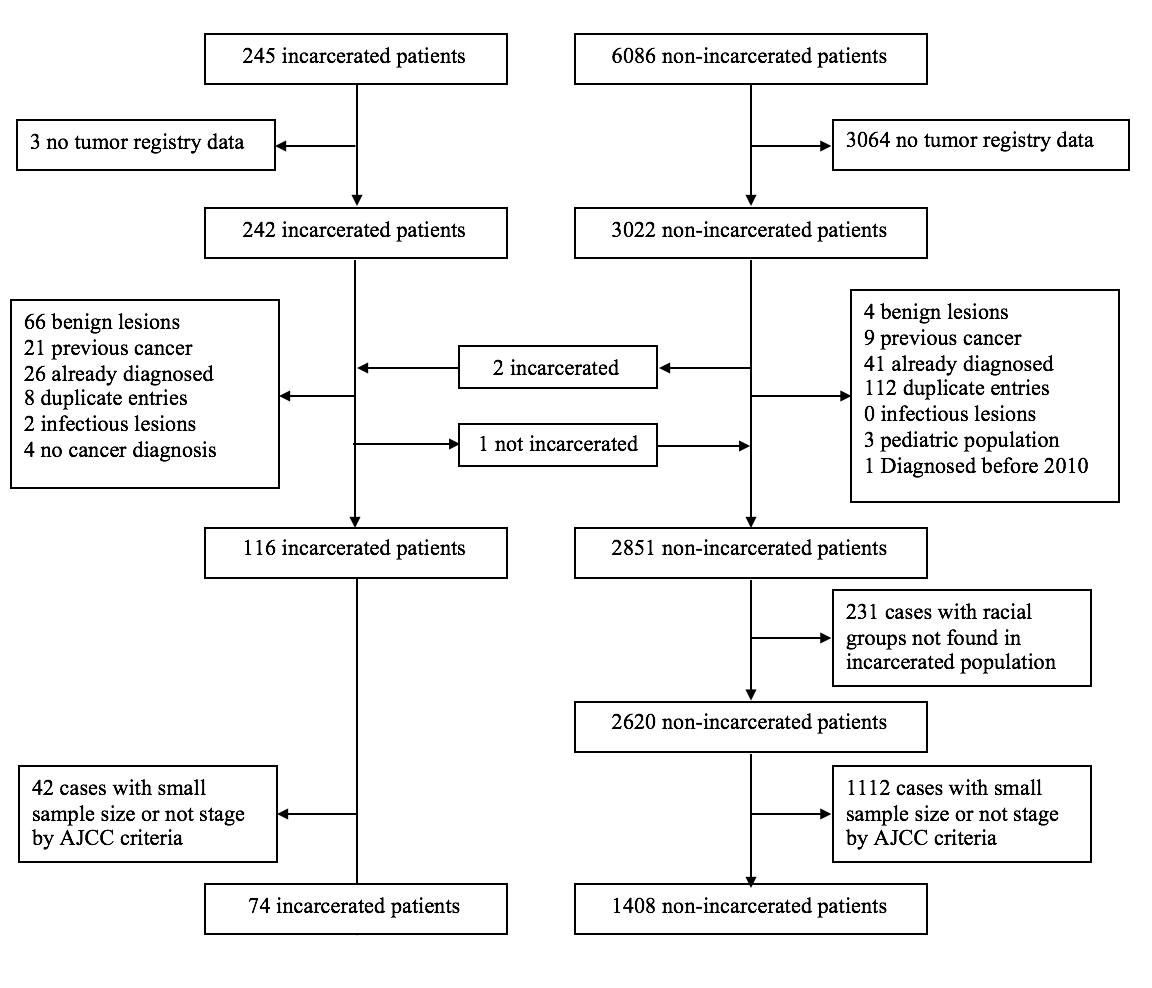

Supplement: S1 Fig — (DOCX) [file pone.0237439.s001.docx]
