# Supplementary material for: Cancer stage at presentation for incarcerated patients at a single urban tertiary care center
Source: PLoS One. 2020 Sep 15;15(9):e0237439. doi: 10.1371/journal.pone.0237439 (PMC7491712; doi:10.1371/journal.pone.0237439)
Supplement: S1 Table — (DOCX) [file pone.0237439.s002.docx]

**S1 Table. Frequency of Cancer Subtypes in incarcerated and non-incarcerated population**

| **Malignancy** | **Non-incarcerated n (%)** | **Incarcerated n (%)** |
| --- | --- | --- |
| Oropharyngeal | 351 (24.9) | 11 (14.9) |
| Bronchopulmonary | 314 (22.3) | 15 (20.3) |
| Hepatobiliary | 67 (4.8) | 23 (31.1) |
| Esophageal | 70 (5.0) | 6 (8.1) |
| Colorectal | 198 (14.1) | 7 (9.5) |
| Prostate | 296 (21.1) | 8 (10.8) |
| Skin | 112 (8.0) | 4 (5.4) |
