# Supplementary material for: Cancer stage at presentation for incarcerated patients at a single urban tertiary care center
Source: PLoS One. 2020 Sep 15;15(9):e0237439. doi: 10.1371/journal.pone.0237439 (PMC7491712; doi:10.1371/journal.pone.0237439)
Supplement: S2 Table — (DOCX) [file pone.0237439.s003.docx]

**S2 Table. Descriptive statistics of cancer staging by incarceration status with confidence intervals**

**Notes:** The table displays unadjusted averages and differences in tumor staging between prisoners and non-prisoners. Screenable cancers include liver, lung, colorectal, and prostate. Differences were assessed using two-sided t-tests. *p<0.05 **p<0.01 ***p<0.001

| **Cancer Type** | **Incarcerated (#)** | | **Clinical Stage** | | | | |  |  |  |  |  |
| --- | --- | --- | --- | --- | --- | --- | --- | --- | --- | --- | --- | --- |
|  |  |  | **T** | | | |  | **N** | | | |  |
|  | **No** | **Yes** | **Incarcerated** | | **Diff** | **P-value** | **95% CI** | **Incarcerated** | | **Diff** | **P-value** | **95% CI** |
|  |  |  | **Yes** | **No** |  |  |  | **Yes** | **No** |  |  |  |
| Oropharyngeal | 351 | 11 | 2.73 | 2.56 | 0.17 | 0.708 | (-0.79, 1.13) | 1.36 | 0.92 | 0.45 | 0.184 | (-0.25, 1.14) |
| Lung | 314 | 15 | 2.67 | 2.52 | 0.15 | 0.675 | (-0.58, 0.87) | 1.13 | 1.22 | -0.08 | 0.808 | (-0.81, 0.65) |
| Liver | 67 | 23 | 2.17 | 1.97 | 0.20 | 0.286 | (-0.18, 0.58) | 0.14 | 0.18 | -0.05 | 0.631 | (-0.23, 0.14) |
| Esophageal | 70 | 6 | 1.67 | 2.26 | -0.60 | 0.139 | (-1.45, 0.26) | 0.83 | 0.72 | 0.11 | 0.801 | (-0.92, 1.14) |
| Colorectal | 198 | 7 | 3.29 | 2.37 | 0.92* | 0.017 | (0.22, 1.62) | 1.14 | 0.57 | 0.57 | 0.261 | (-0.55, 1.7) |
| Adenocarcinoma of the prostate | 296 | 8 | 1.75 | 1.94 | -0.19 | 0.488 | (-0.78, 0.41) | 0.12 | 0.13 | 0.00 | 0.997 | (-0.3, 0.3) |
| Skin | 112 | 4 | 2.00 | 2.02 | -0.02 | 0.977 | (-2.38, 2.34) | 1.00 | 0.25 | 0.75 | 0.532 | (-3.52, 5.02) |
| Screenable Cancers | 875 | 53 | 2.46 | 2.30 | 0.16 | 0.189 | (-0.08, 0.4) | 0.69 | 0.67 | 0.02 | 0.828 | (-0.18, 0.23) |
| Overall | 1408 | 74 | 2.37 | 2.31 | 0.06 | 0.618 | (-0.19, 0.32) | 0.72 | 0.67 | 0.05 | 0.670 | (-0.2, 0.3) |

| **Cancer Type** |  |  |  | **Clinical Stage** | | | | |  |  |
| --- | --- | --- | --- | --- | --- | --- | --- | --- | --- | --- |
|  | **M** | | | |  | **AJCC** | | | |  |
|  | **Incarcerated** | | **Diff** | **P-value** | **95% CI** | **Incarcerated** | | **Diff** | **P-value** | **95% CI** |
|  | **Yes** | **No** |  |  |  | **Yes** | **No** |  |  |  |
| Oropharyngeal | 0.00 | 0.02 | -0.02* | 0.014 | (-0.03, 0) | 3.45 | 3.00 | 0.46 | 0.273 | (-0.42, 1.33) |
| Lung | 0.47 | 0.46 | 0.00 | 0.979 | (-0.29, 0.29) | 2.87 | 2.96 | -0.09 | 0.786 | (-0.83, 0.64) |
| Liver | 0.09 | 0.19 | -0.11 | 0.172 | (-0.26, 0.05) | 2.30 | 2.21 | 0.10 | 0.704 | (-0.41, 0.6) |
| Esophageal | 0.33 | 0.31 | 0.02 | 0.933 | (-0.52, 0.56) | 2.50 | 2.73 | -0.23 | 0.707 | (-1.67, 1.21) |
| Colorectal | 0.29 | 0.31 | -0.03 | 0.884 | (-0.48, 0.42) | 2.86 | 2.58 | 0.27 | 0.462 | (-0.56, 1.11) |
| Adenocarcinoma of the prostate | 0.12 | 0.11 | 0.01 | 0.938 | (-0.29, 0.31) | 2.38 | 2.39 | -0.01 | 0.961 | (-0.64, 0.61) |
| Skin | 0.00 | 0.04 | -0.04* | 0.045 | (-0.07, 0.00) | 1.75 | 1.60 | 0.15 | 0.774 | (-1.34, 1.65) |
| Screenable Cancers | 0.19 | 0.21 | -0.01 | 0.714 | (-0.09, 0.06) | 2.78 | 2.63 | 0.15 | 0.203 | (-0.08, 0.38) |
| Overall | 0.19 | 0.20 | -0.01 | 0.786 | (-0.11, 0.08) | 2.64 | 2.64 | -0.01 | 0.964 | (-0.28, 0.27) |
