# Supplementary material for: Cancer stage at presentation for incarcerated patients at a single urban tertiary care center
Source: PLoS One. 2020 Sep 15;15(9):e0237439. doi: 10.1371/journal.pone.0237439 (PMC7491712; doi:10.1371/journal.pone.0237439)
Supplement: S3 Table — (DOCX) [file pone.0237439.s004.docx]

**S3 Table. Descriptive statistics for early vs. late cancer staging, by cancer subtype**

**Notes:** The table displays unadjusted averages and differences in tumor staging between prisoners and non-prisoners. Screenable cancers include liver, lung, colorectal, and prostate. Differences were assessed using two-sided t-tests. *p<0.05 **p<0.01 ***p<0.001

| **Cancer Type** | **Incarcerated (#)** | | **Late Diagnosis** | | | | | | | | | | | | | | |
| --- | --- | --- | --- | --- | --- | --- | --- | --- | --- | --- | --- | --- | --- | --- | --- | --- | --- |
|  |  |  | **T** | | | | | **N** | | | | | **AJCC** | | | | |
|  | **No** | **Yes** | **Incarcerated** | | **Diff** | **P-value** | **95% CI** | **Incarcerated** | | **Diff** | **P-value** | **95% CI** | **Incarcerated** | | **Diff** | **P-value** | **95% CI** |
|  |  |  | **Yes** | **No** |  |  |  | **Yes** | **No** |  |  |  | **Yes** | **No** |  |  |  |
| Oropharyngeal | 351 | 11 | 0.55 | 0.49 | 0.06 | 0.720 | (-0.29, 0.41) | 0.73 | 0.50 | 0.23 | 0.142 | (-0.09, 0.54) | 0.82 | 0.67 | 0.15 | 0.261 | (-0.13, 0.42) |
| Lung | 314 | 15 | 0.53 | 0.46 | 0.07 | 0.611 | (-0.22, 0.36) | 0.47 | 0.57 | -0.10 | 0.460 | (-0.39, 0.19) | 0.67 | 0.70 | -0.04 | 0.774 | (-0.31, 0.24) |
| Liver | 67 | 23 | 0.35 | 0.33 | 0.01 | 0.902 | (-0.22, 0.25) | 0.14 | 0.15 | -0.02 | 0.863 | (-0.19, 0.16) | 0.30 | 0.37 | -0.07 | 0.552 | (-0.3, 0.16) |
| Esophageal | 70 | 6 | 0.17 | 0.50 | -0.33 | 0.106 | (-0.76, 0.09) | 0.50 | 0.59 | -0.09 | 0.699 | (-0.67, 0.48) | 0.50 | 0.59 | -0.09 | 0.724 | (-0.66, 0.49) |
| Colorectal | 198 | 7 | 0.86 | 0.52 | 0.34 | 0.055 | (-0.01, 0.69) | 0.57 | 0.41 | 0.16 | 0.455 | (-0.33, 0.66) | 0.57 | 0.55 | 0.02 | 0.908 | (-0.47, 0.52) |
| Adenocarcinoma of the prostate | 296 | 8 | 0.12 | 0.17 | -0.04 | 0.736 | (-0.34, 0.25) | 0.12 | 0.12 | 0.01 | 0.941 | (-0.29, 0.31) | 0.25 | 0.24 | 0.01 | 0.969 | (-0.38, 0.39) |
| Skin | 112 | 4 | 0.33 | 0.34 | -0.01 | 0.987 | (-1.4, 1.39) | 0.33 | 0.13 | 0.20 | 0.604 | (-1.21, 1.62) | 0.25 | 0.17 | 0.08 | 0.767 | (-0.7, 0.87) |
| Screenable Cancers | 875 | 53 | 0.47 | 0.39 | 0.07 | 0.159 | (-0.03, 0.17) | 0.37 | 0.38 | -0.01 | 0.767 | (-0.11, 0.08) | 0.53 | 0.51 | 0.02 | 0.631 | (-0.08, 0.12) |
| Overall | 1408 | 74 | 0.42 | 0.40 | 0.03 | 0.665 | (-0.09, 0.14) | 0.38 | 0.38 | 0.00 | 0.944 | (-0.12, 0.11) | 0.49 | 0.51 | -0.03 | 0.673 | (-0.14, 0.09) |
