# Supplementary material for: Cancer stage at presentation for incarcerated patients at a single urban tertiary care center
Source: PLoS One. 2020 Sep 15;15(9):e0237439. doi: 10.1371/journal.pone.0237439 (PMC7491712; doi:10.1371/journal.pone.0237439)
Supplement: S4 Table — (DOCX) [file pone.0237439.s005.docx]

**S4 Table. Adjusted regression results for the effects of incarceration status on initial cancer staging, by cancer subtype with confidence intervals**

**Notes:** The table displays average differences in tumor staging between prisoners and non-prisoners after inverse probability of treatment weighting. Screenable cancers include liver, lung, colorectal, and prostate. p<0.05 **p<0.01 ***p<0.001

| **Cancer Type** | **Incarcerated (#)** | | **Clinical Stage** | | | | | | | | | | |  |
| --- | --- | --- | --- | --- | --- | --- | --- | --- | --- | --- | --- | --- | --- | --- |
|  | **No** | **Yes** | **T** | | | **N** | | | **M** | |  | **AJCC** | |  |
|  |  |  | **Diff** | **P** | **95% CI** | **Diff** | **P** | **95% CI** | **Diff** | **P** | **95% CI** | **Diff** | **P** | **95% CI** |
| Oropharyngeal | 351 | 11 | 0.16 | 0.328 | (-0.16, 0.48) | 0.37** | 0.003 | (0.12, 0.61) | -0.01 | 0.304 | (-0.04, 0.01) | 0.36* | 0.025 | (0.05, 0.67) |
| Lung | 314 | 15 | 0.19 | 0.181 | (-0.09, 0.46) | 0.03 | 0.810 | (-0.24, 0.30) | 0.06 | 0.310 | (-0.05, 0.17) | 0.03 | 0.844 | (-0.24, 0.30) |
| Lung w/ additional risk factors | 79 | 14 | -0.11 | 0.662 | (-0.61, 0.39) | 0.60* | 0.018 | (0.11, 1.10) | 0.08 | 0.458 | (-0.13, 0.28) | 0.18 | 0.433 | (-0.27, 0.67) |
| Liver | 67 | 23 | 0.21 | 0.311 | (-0.20, 0.63) | -0.03 | 0.780 | (-0.26, 0.20) | -0.07 | 0.444 | (-0.24, 0.11) | 0.11 | 0.702 | (-0.47, 0.70) |
| Liver w/ additional risk factors | 41 | 23 | 0.35 | 0.078 | (-0.04, 0.73) | -0.10 | 0.148 | (-0.25, 0.04) | -0.01 | 0.873 | (-0.16, 0.14) | 0.27 | 0.330 | (-0.28, 1.67) |
| Esophageal | 70 | 6 | -0.39 | 0.071 | (-0.81, 0.03) | 0.25 | 0.153 | (-0.09, 0.59) | 0.05 | 0.641 | (-0.17, 0.28) | 0.07 | 0.792 | (-0.44, 1.67) |
| Colorectal | 198 | 7 | 0.93*** | 0.000 | (0.59, 1.28) | 0.48*** | 0.000 | (0.22, 0.73) | -0.06 | 0.437 | (-0.21, 0.09) | 0.27 | 0.144 | (-0.09, 0.67) |
| Adenocarcinoma of the prostate | 296 | 8 | -0.12 | 0.263 | (-0.33, 0.09) | 0.02 | 0.743 | (-0.08, 0.10) | 0.04 | 0.384 | (-0.04, 0.11) | 0.04 | 0.712 | (-0.15, 7.67) |
| Skin | 112 | 4 | 0.22 | 0.453 | (-0.36, 0.80) | 0.59* | 0.014 | (0.12, 1.05) | -0.06 | 0.221 | (-0.15, 0.03) | 0.06 | 0.778 | (-0.34, 0.67) |
| Screenable | 875 | 53 | 0.23** | 0.002 | (0.09, 0.38) | 0.17** | 0.008 | (0.04, 0.29) | -0.04 | 0.219 | (-0.10, 0.02) | 0.04 | 0.573 | (-0.11, 0.67) |
| Overall | 1408 | 74 | 0.14* | 0.033 | (0.01, 0.26) | 0.23*** | 0.000 | (0.13, 0.34) | -0.04 | 0.087 | (-0.08, 0.01) | 0.09 | 0.139 | (-0.03, 0.67) |
