# Supplementary material for: Cancer stage at presentation for incarcerated patients at a single urban tertiary care center
Source: PLoS One. 2020 Sep 15;15(9):e0237439. doi: 10.1371/journal.pone.0237439 (PMC7491712; doi:10.1371/journal.pone.0237439)
Supplement: S5 Table — (DOCX) [file pone.0237439.s006.docx]

**S5 Table. Adjusted regression results for the effects of incarceration status on early vs. late cancer staging, by cancer subtype**

**Notes:** The table displays average differences in the proportion prisoners and non-prisoners who were diagnosed at a later stage (defined as tumor stage 3+, nodal stage 1+, and AJCC stages 3+) after inverse probability of treatment weighting. Screenable cancers include liver, lung, colorectal, and prostate. *p<0.05 **p<0.01 ***p<0.001

| **Cancer Type** | **Incarcerated (#)** | | **Late Diagnosis** | | | | | | | | |
| --- | --- | --- | --- | --- | --- | --- | --- | --- | --- | --- | --- |
|  | **No** | **Yes** | **T** | | | **N** | | | **AJCC** | | |
|  |  |  | **Diff** | **P** | **95% CI** | **Diff** | **P** | **95% CI** | **Diff** | **P** | **95% CI** |
| Oropharyngeal | 351 | 11 | 0.08 | 0.23 | (-0.05, 0.20) | 0.22*** | 0.00 | (0.10, 0.34) | 0.09 | 0.11 | (-0.02, 0.20) |
| Lung | 314 | 15 | 0.08 | 0.17 | (-0.03, 0.19) | -0.07 | 0.25 | (-0.18, 0.05) | -0.01 | 0.91 | (-0.11, 0.10) |
| Lung w/ additional risk factors | 79 | 14 | 0.06 | 0.61 | (-0.16, 0.27) | 0.09 | 0.35 | (-0.11, 0.29) | 0.10 | 0.24 | (-0.07,0.28) |
| Liver | 67 | 23 | 0.02 | 0.92 | (-0.27, 0.30) | -0.02 | 0.86 | (-0.24, 0.20) | -0.06 | 0.67 | (-0.34, 0.22) |
| Liver w/ additional risk factors | 41 | 23 | 0.21 | 0.05 | (0.00, 0.42) | -0.10 | 0.15 | (-0.25, 0.04) | 0.19 | 0.11 | (-0.05,0.41) |
| Esophageal | 70 | 6 | -0.261* | 0.03 | (-0.49, -0.03) | -0.03 | 0.78 | (-0.24, 0.18) | 0.04 | 0.69 | (-0.17,0.26) |
| Colorectal | 198 | 7 | 0.34*** | 0.00 | (0.19, 0.49) | 0.16* | 0.04 | (0.01, 0.31) | 0.06 | 0.48 | (-0.10, 0.21) |
| Adenocarcinoma of the prostate | 296 | 8 | -0.03 | 0.51 | (-0.13, 0.07) | 0.02 | 0.69 | (-0.06, 0.10) | 0.03 | 0.56 | (-0.08,0.14) |
| Skin | 112 | 4 | 0.02 | 0.88 | (-0.24, 0.28) | 0.12 | 0.23 | (-0.07, 0.30) | -0.03 | 0.74 | (-0.19,0.14) |
| Screenable | 875 | 53 | 0.07* | 0.04 | (0.00, 0.14) | 0.02 | 0.47 | (-0.04, 0.08) | 0.02 | 0.62 | (-0.05,0.08) |
| Overall | 1408 | 74 | 0.03 | 0.38 | (-0.03, 0.08) | 0.05 | 0.05 | (0.00, 0.10) | 0.01 | 0.82 | (-0.02, 0.08) |
