# Supplementary material for: Cancer stage at presentation for incarcerated patients at a single urban tertiary care center
Source: PLoS One. 2020 Sep 15;15(9):e0237439. doi: 10.1371/journal.pone.0237439 (PMC7491712; doi:10.1371/journal.pone.0237439)
Supplement: S6 Table — (DOCX) [file pone.0237439.s007.docx]

**S6 Table. Lung Cancer Risk Factors in IP and NIP groups**

**Notes**: The table displays frequencies of select risk factors for prisoners and non-prisoners diagnosed with lung cancers. Differences are assessed using t-tests (for continuous variables) or chi-square tests (for categorical variables). Percentages may not sum to 100 due to rounding. p<0.05 **p<0.01 ***p<0.001.

| **Variable** | **Not incarcerated** | **Incarcerated** | **P-value** |
| --- | --- | --- | --- |
| N | 79 | 14 |  |
| Race (%) |  |  | 0.037* |
| African American | 42 (53.1) | 3 (21.4) |  |
| Hispanic | 4 (5.1) | 0 (0.0) |  |
| Caucasian | 33 (41.8) | 11 (78.6) |  |
| Sex = M (%) | 43 (54.4) | 13 (92.9) | 0.023* |
| Age (mean (sd)) | 62.7 (9.7) | 63.4 (8.5) | 0.789 |
| Type of Lung Cancer (%) |  |  | 0.505 |
| Lung, NOS | 5 (6.3) | 1 ( 7.1) |  |
| Squamous Cell | 17 (21.5) | 1 ( 7.1) |  |
| Small Cell | 14 (17.7) | 2 (14.3) |  |
| Adenocarcinoma | 39 (49.4) | 9 (64.3) |  |
| Large Cell | 1 (1.3) | 1 (7.1) |  |
| Mesothelioma | 3 (3.8) | 0 ( 0.0) |  |
| Smoking status (%) |  |  | <.001*** |
| Current | 53 (63.1) | 0 (0.0) |  |
| Former < 15 years | 16 (19.0) | 6 (42.9) |  |
| Former > 15 years | 10 (11.9) | 7 (50.0) |  |
| Never | 5 (6.0) | 1 (7.1) |  |
| Pack years (mean (sd)) | 41.0 (27.0) | 49.4 (39.4) | 0.456 |
| Asbestos Exposure (%) |  |  | 0.736 |
| Yes | 7 (8.3) | 2 (14.3) |  |
| No | 16 (19.0) | 2 (14.3) |  |
| Unknown | 61 (72.6) | 10 (71.4) |  |
| TB Exposure |  |  | 0.786 |
| Yes | 12 (14.3) | 3 (21.4) |  |
| No | 14 (16.7) | 2 (14.3) |  |
| Unknown | 58 (69.0) | 9 (64.3) |  |
| Family history of lung cancer |  |  | 0.269 |
| Yes | 12 (14.3) | 4 (28.6) |  |
| No | 55 (65.5) | 9 (64.3) |  |
| Unknown | 17 (20.2) | 1 (7.1) |  |
| T (%) |  |  | 0.896 |
| 0 | 1 (1.3) | 0 (0.0) |  |
| 1 | 11 (14.1) | 3 (21.4) |  |
| 2 | 25 (32.1) | 3 (21.4) |  |
| 3 | 10 (12.8) | 2 (14.3) |  |
| 4 | 31 (39.7) | 6 (42.9) |  |
| N (%) |  |  | 0.665 |
| 0 | 30 (38.0) | 7 (50.0) |  |
| 1 | 5 (6.3) | 0 (0.0) |  |
| 2 | 29 (36.7) | 4 (28.6) |  |
| 3 | 15 (19.0) | 3 (21.4) |  |
| M (%) |  |  | 0.848 |
| 0 | 34 (43.0) | 7 (50.0) |  |
| 1 | 45 (57.0) | 7 (50.0) |  |
| AJCC (%) |  |  | 0.914 |
| 1 | 12 (15.2) | 3 (21.4) |  |
| 2 | 5 (6.3) | 1 (7.1) |  |
| 3 | 15 (19.0) | 3 (21.4) |  |
| 4 | 47 (59.5) | 7 (50.0) |  |
