# Supplementary material for: Cancer stage at presentation for incarcerated patients at a single urban tertiary care center
Source: PLoS One. 2020 Sep 15;15(9):e0237439. doi: 10.1371/journal.pone.0237439 (PMC7491712; doi:10.1371/journal.pone.0237439)
Supplement: S7 Table — (DOCX) [file pone.0237439.s008.docx]

**S7 Table. Hepatocellular Carcinoma Risk Factors in IP and NIP groups**

**Notes**: The table displays frequencies of select risk factors for prisoners and non-prisoners diagnosed with hepatocellular carcinoma. Differences are assessed using t-tests (for continuous variables) or chi-square tests (for categorical variables). Percentages may not sum to 100 due to rounding. p<0.05 **p<0.01 ***p<0.001

|  | **Not incarcerated** | **Incarcerated** | **P value** |
| --- | --- | --- | --- |
| N | 41 | 23 |  |
| Race (%) |  |  | 0.447 |
| African American | 16 (39.0) | 6 (26.1) |  |
| Hispanic | 5 (12.2) | 2 (8.7) |  |
| Caucasian | 20 (48.8) | 15 (65.2) |  |
| Sex = M (%) | 41 (100.0) | 23 (100.0) | -- |
| Age (mean (sd)) | 59.9 (9.3) | 59.7 (6.2) | 0.908 |
| HBV Status (%) |  |  | 0.19 |
| Current | 9 (22.0) | 2 (8.7) |  |
| Resolved | 10 (24.4) | 10 (43.5) |  |
| Negative | 22 (53.7) | 11 (47.8) |  |
| HCV Status (%) |  |  | 0.061 |
| Negative | 8 (19.5) | 0 (0.0) |  |
| Positive | 33 (80.5) | 23 (100.0) |  |
| HCV Treated (%) |  |  | 0.871 |
| Yes | 11 (33.3) | 9 (39.1) |  |
| No | 22 (66.7) | 14 (60.9) |  |
| Smoking Status (%) |  |  | 0.152 |
| Current | 18 (43.9) | 5 (21.7) |  |
| Former | 14 (34.1) | 13 (56.5) |  |
| Never | 9 (22.0) | 5 (21.7) |  |
| Cirrhosis (%) |  |  | 0.476 |
| Yes | 38 (92.7) | 23 (100.0) |  |
| No | 3 (7.3) | 0 (0.0) |  |
| History of Alcohol Abuse (%) |  |  | 0.974 |
| Yes | 18 (43.9) | 10 (43.4) |  |
| No | 23 (56.1) | 13 (56.5) |  |
| T (%) |  |  | 0.092 |
| 1 | 19 (46.3) | 4 (17.4) |  |
| 2 | 11 (26.8) | 11 (47.8) |  |
| 3 | 10 (24.4) | 8 (34.8) |  |
| 4 | 1 (2.4) | 0 (0.0) |  |
| N (%) |  |  | 0.495 |
| 0 | 39 (95.1) | 20 (87.0) |  |
| 1 | 2 (4.9) | 3 (13.0) |  |
| M (%) |  |  | 0.889 |
| 0 | 37 (90.2) | 21 (91.3) |  |
| 1 | 4 (9.8) | 2 (8.7) |  |
| AJCC (%) |  |  | 0.075 |
| 1 | 19 (46.3) | 4 (17.4) |  |
| 2 | 11 (26.8) | 12 (52.2) |  |
| 3 | 7 (17.1) | 3 (13.0) |  |
| 4 | 4 (9.8) | 4 (17.4) |  |
