# Supplementary material for: Cancer stage at presentation for incarcerated patients at a single urban tertiary care center
Source: PLoS One. 2020 Sep 15;15(9):e0237439. doi: 10.1371/journal.pone.0237439 (PMC7491712; doi:10.1371/journal.pone.0237439)
Supplement: Attachment — Submitted filename: Response to Reviewers v2.docx [file pone.0237439.s010.docx]

July 17^th^, 2020

Dear Dr. Lim,

Thank you for the opportunity to revise and resubmit our manuscript, “Stage at presentation for incarcerated patients at a single urban tertiary care center,” for consideration at *PLOS ONE.*

The reviewers’ and editors’ comments have been very helpful and strengthened the paper. Our responses are below.

Sincerely,

Kathryn Sunthankar, MD

**Editorial comments**

1. Abstract - You are not examining potential delays, you are examining differences in stage at diagnosis. If you were examining potential delays, you would also be looking individual and system level reasons for delay.

*We thank reviewer #2 for their input and our manuscript now refers to “differences” instead of “delays.”*

2. Page 3, line 45 - At least put that they are not in a position to advocate for themselves first. Really, blaming poor health literacy on the part of prisoners for not getting screened is ridiculous. In almost every situation that I have seen--jails, prisons, county, state, federal, prisoners CANNOT advocate for themselves. I feel as if you are looking at this from only the perspective of a community provider or clinic. I am really trying to be non-judgmental about this but feel as if the whole writing team needs to spend a day shadowing a provider inside of a facility.

*We thank reviewer #2 for their input and agree that these patients are unable to advocate for themselves while incarcerated and this is of great importance. We aimed to summarize the sparse research on etiologies of decreased screening, and did not intend to characterize differences in cancer staging as primarily a failure on the part of the incarcerated patient. The study team has extensive first-hand experience working with incarcerated populations. We agree that they are often limited by what they have available and often there are constrictions on practitioners ability to truly practice standard-of-care medicine with appropriate treatment of conditions (i.e. chemotherapy, hepatitis C treatment), let alone screening for these conditions and other preventative care. We have changed our introduction to now state: “Incarcerated patients (IP) face both systemic barriers to care as well as vulnerability due to impediments to self-advocating due to their status as an inmate. Both these issues may delay or limit their receipt of treatments that – outside the prison system – are more often given, such as direct-acting anti-viral agents for hepatitis C infection and opioid maintenance therapy for substance use disorders.”*

*We have also removed a reference to lower health literacy that was on lines 92-94.*

*We have updated the sentence (line 99-101) to say the following “Together these studies have highlighted that IP are less likely to receive the appropriate screenings compared to the general population.”*

3. Page 4, line 84 - So many qualifiers. Decreased availability, likely, also contribute. Just say it. Lack of availability contributes to lack of screening.

*We have streamlined our introduction to now state: “Multiple reasons for decreased screenings among patients who are currently or previously incarcerated have been suggested, including poorer connection with primary care resources and lack of availability while incarcerated and following incarceration due to systemic barriers patients who were previously incarcerated also face.”*

4. Page 14, line 284 - Encourage? Direct routine screening

*We agree and have changed “encourage” to “direct” as recommended.*
